# A rare case of benign multicystic peritoneal mesothelioma misdiagnosed as hydatid cyst found in the liver parenchyma and abdomen cavity of a male with asbestos exposure

**DOI:** 10.1186/s12876-021-01947-7

**Published:** 2021-10-12

**Authors:** Ahmad Beshr Kelarji, Mohammad Sami Alshutaihi, Ahmad Ghazal, Nihad Mahli, Sarab Agha

**Affiliations:** 1 Department of Pathology, Aleppo University Hospital, Aleppo, Syria; 2 Division of Neurology, Department of Internal Medicine, Aleppo University Hospital, Aleppo, Syria; 3 Surgery Department, Faculty of Medicine, Aleppo University Hospital, University of Aleppo, Aleppo, Syria; 4 Faculty of Medicine, Aleppo University Hospital, University of Aleppo, Aleppo, Syria

**Keywords:** Peritoneum, Benign multicystic mesothelioma, Abdomen, Asbestos, Omentum, Case report

## Abstract

**Background:**

Benign Multicystic Peritoneal Mesothelioma (BMPM) is one of the rarest diseases in medicine with only more than 200 cases worldwide. This paper aims to report a case of Benign Multicystic Peritoneal Mesothelioma that strangely arose from the liver and was long treated as Hydatid cyst. The case also had many risk factors including asbestos exposure that had not yet been linked with Benign Multicystic Peritoneal Mesothelioma.

**Case presentation:**

We report a case of a 62 years old male with a history of a perforated peptic ulcer and a cystic mass in the liver that was misdiagnosed as hydatid cyst 7 years ago. He presented with generalized abdominal pain and bloating. Image studies showed many cystic formations filled with clear fluid. An en bloc surgery was performed and a pathologic study showed a multiloculated mass lined by flat or cuboidal epithelium leading to the diagnosis of BMPM. A follow up was scheduled after 3 months revealed total recurrence.

**Conclusion:**

BMPM resembles many other cystic lesions in the abdomen and should be taken into consideration when dealing with nontypical cystic formations. Its diagnostic and treatment methods are still hazy making this disease difficult to approach.

## Background

Benign multicystic peritoneal mesothelioma is a difficult disease to diagnose due to its rarity on one hand and also to its many similarities (clinically and radiologically) to a lot of other cystic lesions of peritoneum on the other hand. Thus, there is no consensus on its diagnostic tools. Since the discovery of its mesothelial nature by Mennemeyer et al. in 1979, about 200 cases have been reported with several etiologies and risk factors described. At present, this topic is still a controversial issue. There is no standard treatment, although a complete resection is mostly favored.

We report a case of a male who had many proposed predisposing factors including asbestos. He has a huge peritoneal tumor which consists of different sized cysts. The patient had a cyst in the liver that was primarily misdiagnosed as hydatid cysts.

## Case presentation

We report a case of a 62- years -old male with a history of a perforated peptic ulcer 15 years ago and a hepatic cyst 7 years ago which was reported by the patient as a hydatid cyst. The patient used to work as a firefighter. He had neither family nor drug history other than being a heavy smoker (40 pack-year). He presented to the emergency department with generalized abdominal pain for 7 days accompanied by bloating, nausea, vomiting, incomplete constipation, and loss of appetite and weight.

**Fig. 1:**
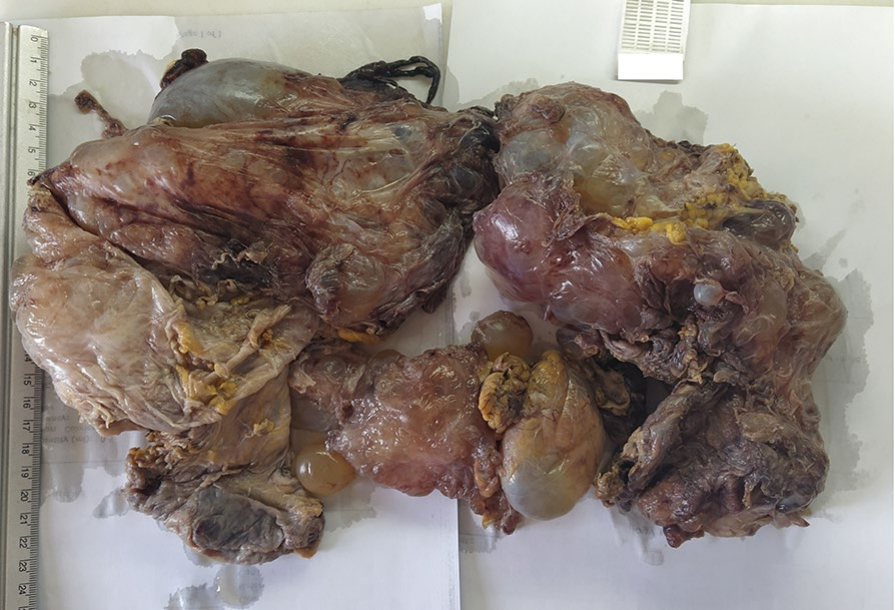
Gross anatomy of the resected mass

On physical examination, the patient looked uncomfortable. Two abdominal hernias were found. Ultrasonography (US) test showed thin walled single layer multicystic formations filling the abdomen. Unfortunately, other imaging tools are not available due to Covid-19 pandemic circumstances. This finding led to the thought that the lesion was a polycystic echinococcosis. However, the Weinberg test came negative. At this point, neoplastic entities include lymphangioma and mesothelioma were considered. Based on the clinical image and the negative results for CEA and CA19-9, Neoplastic mucinous lesions were excluded.

Due to the aggravation of constipation, a palliative and exploratory laparotomy was performed. Multiple cysts in different shapes and sizes were found all over the peritoneum and omentum. The cysts were soft, filled with clear-lucent fluid. However, only large accessible cysts were cut out (en bloc). Oddly, a 10-cm-cyst was found in the parenchyma of the liver’s right lobe.

**Fig. 2:**
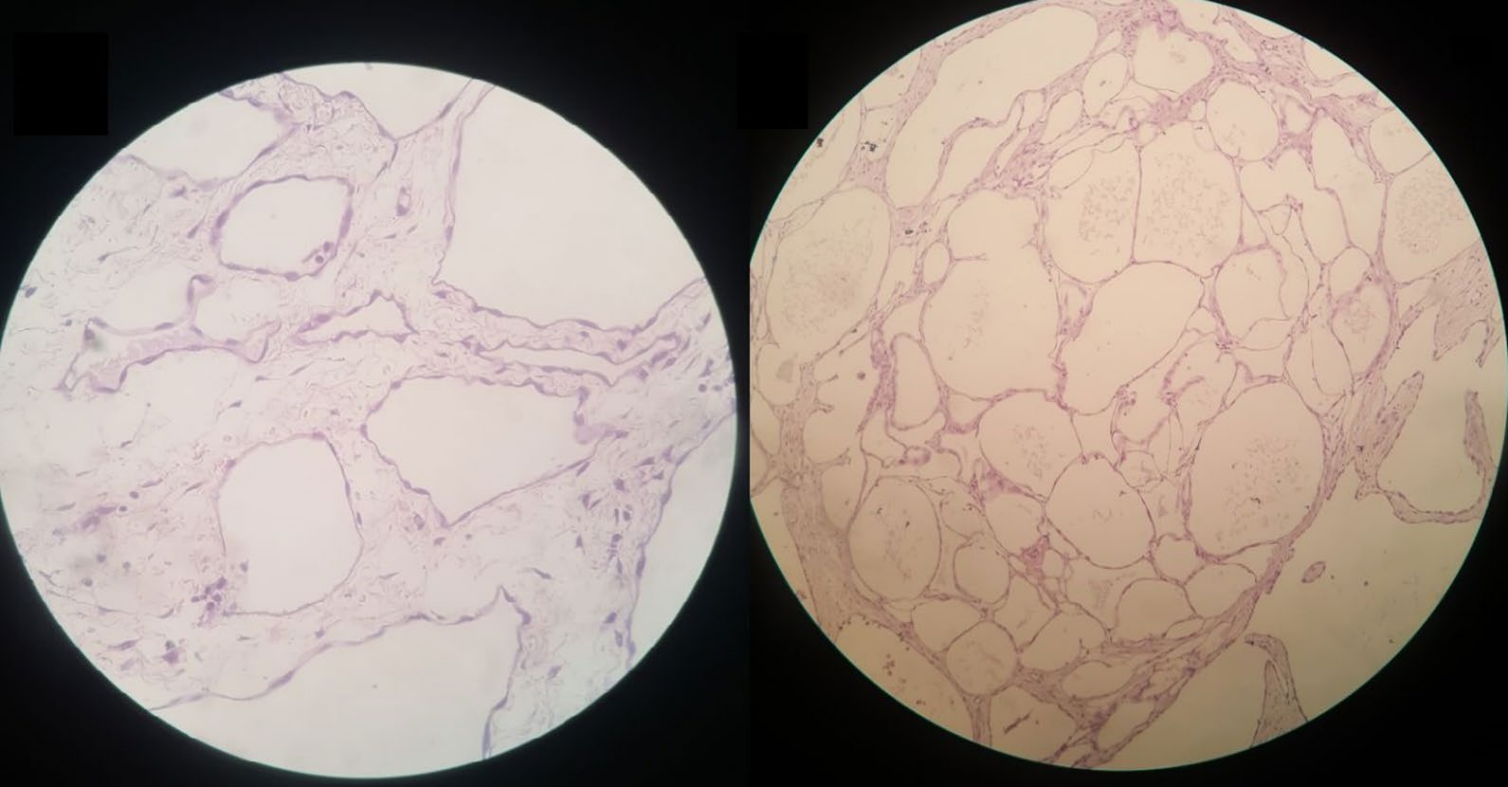
H&E stain of the mass: the figure exhibits multiple cystic cavities with thin fibrous wall lined by flat to cuboidal epithelium and containing eosinophilic material, these cysts are separated by stroma of loose fibrous tissue, without inflammatory infiltrates of surrounding stroma

The pathology exam revealed a multi-cavitated mass measuring (6 * 20 * 30 cm) [Fig. 1] and lined by thin fibrous walls of flat or cuboidal epithelium, containing eosinophilic homogeneous material [Fig. 2]. The cysts are embedded in loose fibrous tissue without inflammatory infiltration of the surrounding stroma.

**Fig. 3:**
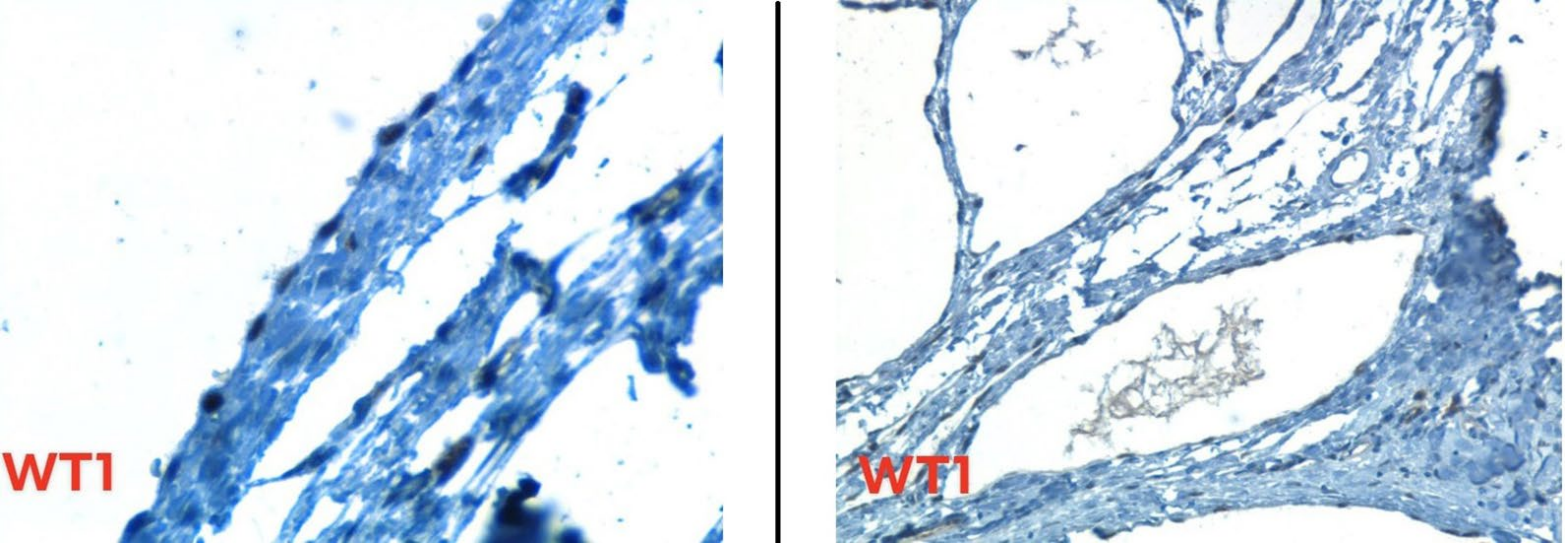
WT1 immunostain is positive in cells lining cystic spaces (positivity is nuclear)

At this level, two differential diagnoses were considered: Cystic Lymphangioma and Benign Multicystic Mesothelioma. The positivity of immunohistochemical markers WT1 and Calretinin,and the negativity of CD34 both affirmed the mesothelial nature of the tumor and led to the diagnosis of BMPM [Fig. 3].

**Fig. 4:**
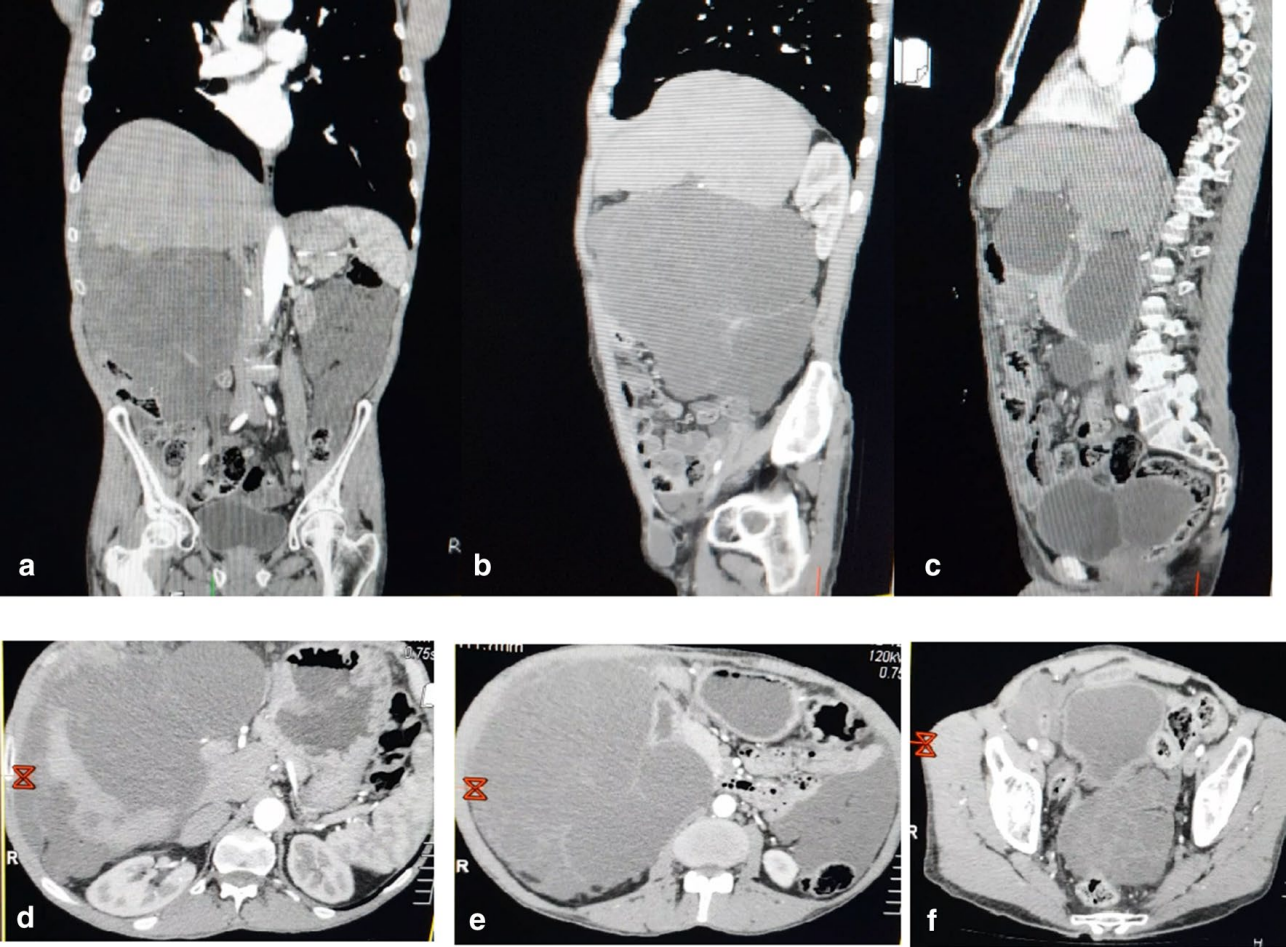
A contrast MSCT of the abdomen and pelvis **A** coronal view, **B** sagittal view, **E** axial view all showing a low dense mass extending from the liver to the pelvis measuring (19.6 * 14.6) cm. **C** sagittal view showing two low dense masses one under the spleen with 15 cm size and the other one is behind the bladder measuring (5 * 9) cm. **D** Axial view of the liver showing a homogenous enhancing area (10 * 7) cm in the right lobe of the liver. **F** Axial view in the pelvis demonstrating a low dense mass pushing the rectum

A contrast CT follow up was scheduled three months after surgery [Fig. 4] and it showed recurrence.

## Discussion and conclusion

Benign Multicystic Peritoneal Mesothelioma (BMPM) is an uncommon disease, with only about 200 cases are reported until 2017 (83% are women) [1]. It was first described by Plaut [1] in 1928, whereas its mesothelial nature was discovered by Mennemeyer and Smith in 1979 [2].

BMPM usually affects the pelvic area. In our case, the tumor started from RUQ (where it was found in the liver parenchyma). To our knowledge, this is just the second case in literature with parenchymal involvement [3].

Five different hypotheses [4] are suggested, the most common one is the inflammatory hypothesis, based on the fact that many cases had a history of surgery or chronic inflammation. Due to the ER, PR presence in some cases [5], the hormone role was studied thoroughly. The other three hypotheses are genetic, embryologic and neoplastic.

Alongside with the differences of theories, different risk factors had been proposed without any confirmation [6] such as surgical history or any chronic inflammatory state especially endometriosis. However, unlike its malignant form or pleural counterpart [7], this type of tumor is not associated with asbestosis exposure [1] although our case seems to be one of few cases in the literature in which the patient had been possibly exposed (the patient used to work as firefighter) [8].

Symptoms of mesothelioma are usually non-specific and mass-related. They include: abdominal pain, tenderness, palpable mass, or weight loss.

Until this day, no imaging or clinical investigations can make the definitive diagnosis. US, CT and MRI can all be used, but MRI remains the best [1].

A biochemical profile for the patient usually has no clinical significance. The affirmative diagnostic tool of this disorder remains excisional biopsy [1]. Under microscope, it appears as translucent serosal cysts of different sizes. The cysts are composed of one layer of flat or cuboidal cells which are embedded in fibrovascular stroma and positive for the mesothelial markers calretinin, the transcription factor WT-1, proliferative protein Ki-67, cytokeratin 5/6,, BAP1,Whereas it is negative for the markers CD 31,CD 34, and factor VIII which favor lymphangioma, the most confusing differential diagnosis [1]. The lymphangioma is a malformation of the lymphatic system. It is a congenital defect although it appears in older ages. It resembles BMPM radiologically and the only way to differentiate between them is by biopsy Other differential diagnoses include; cystic adenomatoïd tumor and malignant peritoneal mesothelioma. The first is a benign entity that arises mainly from the serosal layer of the uterus or epididymis, the other which is malignant, is highly related to asbestosis exposure and it appears as a solid irregular mass. In the light of this, some studies suggest that BMPM is a borderline lesion between the two cases. Pseudomyxoma peritonei (PMP) is another differential diagnoses which is a rare case that arises from predisposing tumors or other lesions and usually causes compression symptoms. In most cases, it takes place after appendiceal carcinoma. Lastly, endometriosis and cystic forms of endosalpingiosis should also be considered as ddx in women [1].

We suggest echinococcus infection as a new DDx. Besides, it's a common finding in developing countries, it shares together with BMPM many aspects clinically and radiologically. On US, both lesions appear as anechoic cysts with liquid content. BMPM is multiloculated and may show a “spider-in-web “sign, whereas the most specific feature of the hydatid cyst is the double echogenic wall. “Snowstorm sign”, “spoked wheel” pattern, “water lily sign” and calcification can also be found depending on the cysts’ level. Unlike US, computed tomography is a useful method for assessing the location and the extent. In case of a simple hydatid cyst, they are both low-density and well-defined masses. MRI as mentioned earlier is considered the best imaging technique. BMPM appear as hypointense lesions on T1 and hyper to intermediate intense on T2, with a mild contrast enhancement of the wall. On the other hand, hydatid cyst has similar intensities.

Until now, there is no precise guideline for BMPM treatment [1]. However, most studies agreed on complete surgical resection alone or with hyperthermic intraperitoneal chemotherapy (a complex of CISPLATIN and DOXORUBICIN) due to the disease tendency to recur. Other treating choices are: hormonal therapy, sclerotherapy, and also potassium-titanyl-phosphate laser vaporization have been mentioned in some recent publications.

Despite being benign, many researchers classified it as a borderline tumor due to its high recurrence rate (50% after 3–27 months) [1] with only two reported cases of malignant transformation [3].

## Limitations

There are some limitations to our case.

1. Our case included a little known lesion, with no proved guideline for diagnosis and treatment.
2. Due to pandemic and war circumstances, it was extremely difficult to conduct all diagnostic tools.
3. Because of limited resources we couldn't make high frequent follow ups for the liver cyst. Thus, reaching the proper diagnosis was delayed.

## Data Availability

All the data supporting our findings is contained within the manuscript.

## Abbreviations

DDx: Differential diagnosis
BMPM: Benign multicystic peritoneal mesothelioma
Us: Ultrasonography
CT: Computed tomography
MRI: Magnetic resonance imaging
RUQ: Right upper quadrant

## References

[1] Noiret B, Renaud F, Piessen G, Eveno C (2019). Multicystic peritoneal mesothelioma: a systematic review of the literature. Pleura Peritoneum.

[2] Mennemeyer R, Smith M (1979). Multicystic, peritoneal mesothelioma: a report with electron microscopy of a case mimicking intra-abdominal cystic hygroma (lymphangioma). Cancer.

[3] Khurram MS, Shaikh H, Khan U, Edens J, Ibrar W, Hamza A, Zaka A, Bano R, Hadid T (2017). Benign multicystic peritoneal mesothelioma: a rare condition in an uncommon gender. Case Rep Pathol.

[4] Cavallaro A, Berretta M, Lo Menzo E, Cavallaro V, Zanghì A, Di Vita M, Cappellani A (2011). Cystic peritoneal mesothelioma: report of a case. Surg Today.

[5] Sawh RN, Malpica A, Deavers MT, Liu J, Silva EG (2003). Benign cystic mesothelioma of the peritoneum: a clinicopathologic study of 17 cases and immunohistochemical analysis of estrogen and progesterone receptor status. Hum Pathol.

[6] Demopoulos RI, Kahn MA, Feiner HD (1986). Epidemiology of cystic mesothelioma. Int J Gynecol Pathol.

[7] Lemen RA (2016). Mesothelioma from asbestos exposures: epidemiologic patterns and impact in the United States. J Toxicol Environ Health Part B Crit Rev.

[8] Kjellevold K, Nesland JM, Holm R, Johannessen JV (1986). Multicystic peritoneal mesothelioma. Pathol Res Pract.

