# Correction to: A rare case of benign multicystic peritoneal mesothelioma misdiagnosed as hydatid cyst found in the liver parenchyma and abdomen cavity of a male with asbestos exposure

**DOI:** 10.1186/s12876-021-02005-y

**Published:** 2021-11-26

**Authors:** Ahmad Beshr Kelarji, Mohammad Sami Alshutaihi, Ahmad Ghazal, Nihad Mahli, Sarab Agha

**Affiliations:** 1 Department of Pathology, Aleppo University Hospital, Aleppo, Syria; 2 Division of Neurology, Department of Internal Medicine, Aleppo University Hospital, Aleppo, Syria; 3 Surgery Department, Faculty of Medicine, Aleppo University Hospital, University of Aleppo, Aleppo, Syria; 4 Faculty of Medicine, Aleppo University Hospital, University of Aleppo, Aleppo, Syria

## Correction to: BMC Gastroenterol (2021) 21:374 10.1186/s12876-021-01947-7

After publication of this article [1], the authors reported that the given and family names of the first 4 authors were incorrectly structured.

The original article [1] has been updated.
